# Laparoscopic Lumen-guided cholecystectomy in face of the difficult gallbladder

**DOI:** 10.1007/s00464-022-09538-7

**Published:** 2022-08-25

**Authors:** James Lucocq, Aaron Taylor, Peter Driscoll, Syed Naqvi, Alasdair MacMillan, Stephen Bennett, Andreas Luhmann, Andrew G. Robertson

**Affiliations:** 1 Department of General and Upper GI Surgery, Victoria Hospital Kirkcaldy, Kirkcaldy, UK; 2 University of Dundee Medical School, Dundee, Scotland

**Keywords:** Laparoscopic cholecystectomy, Salvage techniques, Cystic duct, Cholecystohepatic triangle, Morbidity

## Abstract

**Background:**

Where the critical view of safety cannot be established during cholecystectomy, certain salvage techniques are indicated to reduce the likelihood of bile duct injury. The present study describes a salvage technique termed the “laparoscopic lumen-guided cholecystectomy” (LLC) and reports its peri-operative outcomes.

**Method:**

A summary of the technique is as follows: (1) Hartmann’s pouch is incised and stones are evacuated; (2) the cystic anatomy is inspected from the inside of the gallbladder; (3) the lumen is used to guide retrograde dissection towards the cystic pedicle; (4) cystic duct control is achieved if deemed safe. LLC cases performed between June 2020 and January 2022 in a single health board were included. The operative details and peri-operative outcomes of the technique are reported and compared to cases of similar difficulty where the LLC was not attempted.

**Results:**

LLC was performed in 4.6% (27/587) of cases. In all 27 cases, LLC was performed for a “frozen” cholecystohepatic triangle. Hartmann’s pouch was completely excised in all cases (27/27) and cystic duct control was achieved in 85.2% of cases (23/27). No cases of bile leak or ductal injury were reported. Rates of bile leak, post-operative complications and ERCP were lower following LLC compared to the group where LLC was not attempted (*p *< 0.01).

**Conclusion:**

LLC is a safe salvage technique and should be considered in cases where the critical view of safety cannot be established. The technique achieves cystic duct control in the majority of cases and favourable outcomes in the face of a difficult cholecystectomy.

Bile duct injury (BDI) is a rare complication (0.3–1.5%) of laparoscopic cholecystectomy (LC) resulting in high rates of morbidity and mortality [1, 2]. The risks of BDI are reduced by achieving the Critical View of Safety (CVS) [3, 4]. In the difficult cholecystectomy where the CVS cannot be established, the operating surgeon may perform “salvage techniques” such as subtotal cholecystectomy, fundus-first approach or conversion-to-open.

Salvage techniques each have their own limitations. Subtotal cholecystectomy does not guarantee cystic duct control and leaves a gallbladder remnant, risking bile leak, retained stones and the need for post-operative endoscopic retrograde cholangiopancreatography (ERCP) [5–7]. Fundus-first approach reportedly has a high rate of ductal injury and of course conversion-to-open is associated with the risks of open surgery and prolonged post-operative recovery [8, 9].

The aim of this study is to describe an alternative salvage technique termed the “laparoscopic lumen-guided cholecystectomy” (LLC), used in cases of a frozen cholecystohepatic triangle where the CVS cannot be established and to report the peri-operative outcomes. The secondary aim was to compare outcomes of LLC to non-LLC in cases of similar difficulty.

## Method

### Population cohort

LLC performed for biliary pathology across a single health board between June 2020 and January 2022 were included in the study. The operations were performed by six Upper GI and HPB surgical consultants across a defined geographical region with a population of approximately 400,000 people.

### Data analysis

Retrospective analysis of prospectively collected data for all cases of LLC was conducted. Pre-operative details were recorded and included demographics, American society of anaesthesiology score (ASA), number of admissions, indication and pre-operative intervention. The operative details were recorded and included intra-operative findings, ‘Nassar’ grade, use of drains and intra-operative complications [10]. Patients were followed up for a median time of 330 days for length of stay, post-operative complications, imaging, intervention (e.g. ERCP, return to theatre) and readmission. As a retrospective review of anonymised data no ethical approval was required by our health board.

### LLC vs. non-LLC

Peri-operative outcomes of LLC were compared to a “non-LLC group” in cases of similar difficulty where the LLC technique was not attempted. To ensure the non-LLC group were of comparable difficulty, all remaining Nassar IV/V gallbladders with a frozen cholecystohepatic triangle over the time period were included as the “non-LLC group”. Overall the LLC and non-LLC cholecystectomies represent the most difficult cholecystectomies performed over the study period. To ensure these two groups were comparable, the pre-operative and operative findings were compared between the two groups.

Peri-operative outcomes were compared using chi squared analysis. A *p*-value < 0.05 was regarded as statistically significant.

### Operative technique

In our centre, the standard operative set-up is a four-port technique in the ‘American’ position. Access is established using the modified open technique using an infraumbilical 12 mm incision. A 10 mm epigastric port is inserted under vision followed by 5 mm right sub-costal and right flank ports. Omental/bowel adhesions to the abdominal wall, obscuring the view of the operating field or restricting operating instruments is divided at the level of the parietal peritoneum. Adhesions between abdominal viscera and the gallbladder or liver are then divided using either sharp or blunt dissection. In cases of suspected empyema or mucocele, the gallbladder is decompressed with a “Veress” needle to aid retraction. The fundus of the gallbladder is retracted towards the right shoulder with a grasper to expose the cholecystohepatic triangle. An initial attempt is made to establish the critical view of safety until deemed unsafe.

**Fig. 1:**
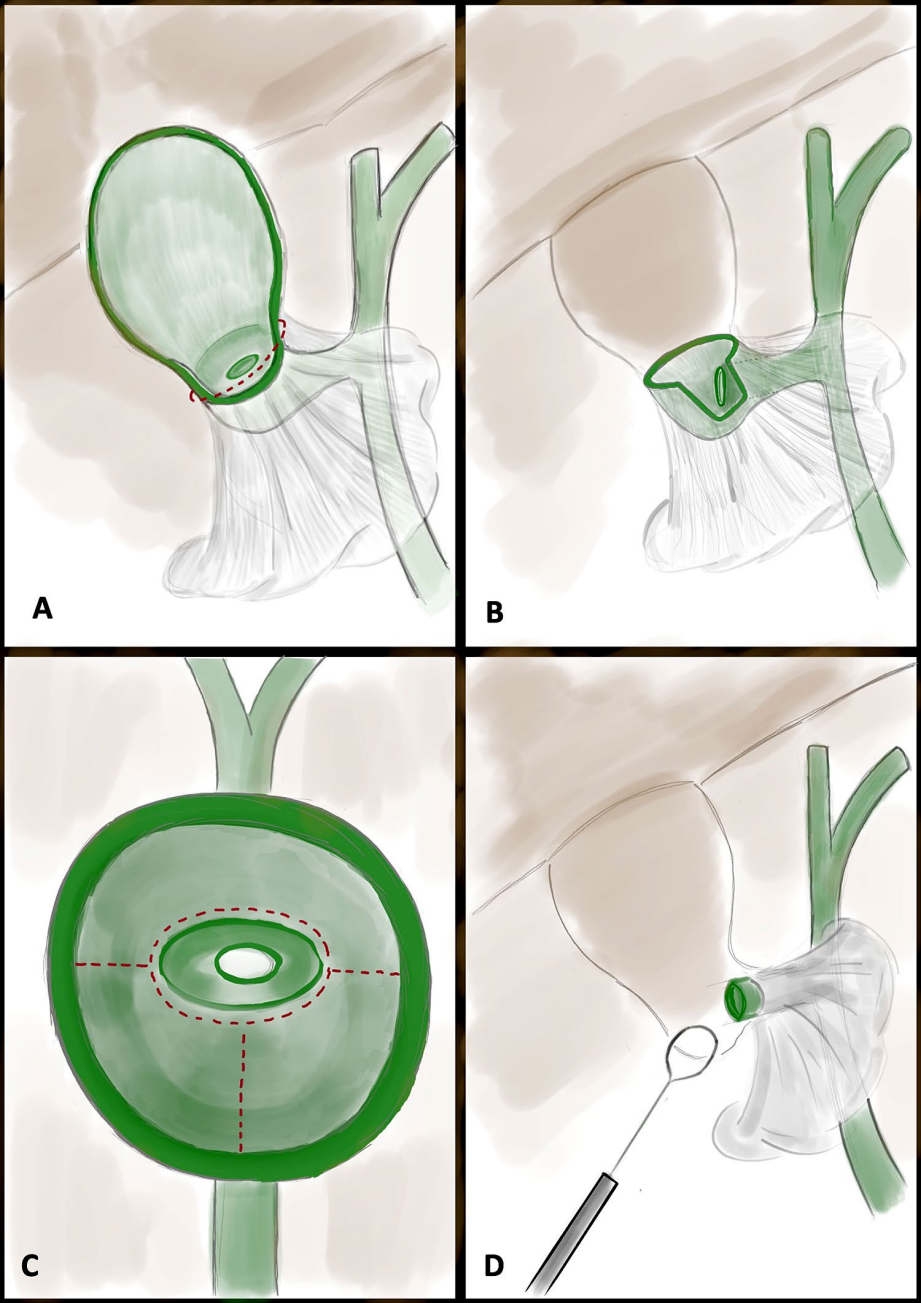
Illustrations of the operative technique. **A** Transverse incision at level of Hartmann’s pouch to gain access to the lumen of the gallbladder where the cystic pedicle cannot be dissected safely; **B** The cystic duct is visible from inside, retrograde dissection frees Hartmann’s pouch from adjacent structures and portions of the anterior wall can be excised to progress towards the cystic duct; **C** luminal view used to guide further retrograde dissection towards the cystic duct; **D** cystic duct control achieved (e.g. with endoloop)

In cases of an LLC, the anterior wall of the gallbladder is incised at the level of Hartmann’s pouch with a transverse incision using hook diathermy and the gallbladder lumen is entered (Fig. 1). Upon entering the gallbladder, intravenous antibiotics (Amoxicillin, Metronidazole and Gentamicin) are administered as a once-off dose in accordance with our local guidelines for intra-abdominal sepsis, unless the patient had already been started on intravenous antibiotics for cholecystitis. Gallbladder stones are removed into a retrieval bag and bile is suctioned until its lumen is cleared. The lumen is inspected using a 30-degree laparoscope to delineate the cystic ductal anatomy to guide dissection in a retrograde direction towards the cystic duct (Fig. 1). To improve the internal view of the cystic duct the gallbladder may be carefully manipulated using a retractor on the gallbladder fundus and on the free edge of the anterior wall. Where the view is still difficult to establish, part of the anterior wall of Hartmann’s can be excised. Retrograde dissection may be required before portions of the anterior wall can be excised.

Both the luminal and external view are used to guide safe retrograde dissection of Hartmann’s pouch by gaining an appreciation of the boundaries of Hartmann’s pouch. A plane along the gallbladder wall is developed using a combination of suction dissection, blunt dissection using a curved Maryland and hook diathermy. Traction on the free edge of Hartmann’s pouch in a superior or lateral direction often facilitates the development of this plane. As retrograde dissection progresses, portions of the anterior wall can be excised to improve the luminal view of the duct and to advance towards the cystic duct. Dissection proceeds until the superior aspect of cystic duct is reached at which point control can be attempted with either endoloops, vicryl ties or “hem-o-loks” if considered safe.

Following this the remaining portions of Hartmann’s pouch can be excised. Retaining portions of anterior wall until cystic duct control offers points for traction, whereas excision of the anterior wall before achieving cystic duct control can improve the luminal view of the cystic duct. Portions of the posterior wall can be left in situ particularly if its dissection would risk ductal injury or liver tear. Intra-abdominal drains are considered subject to the suspected risk of bile leak and peritonitis. The gallbladder is then retrieved in a retrieval bag through the umbilical port. The umbilical fascia is closed followed by skin closure.

**Fig. 2:**
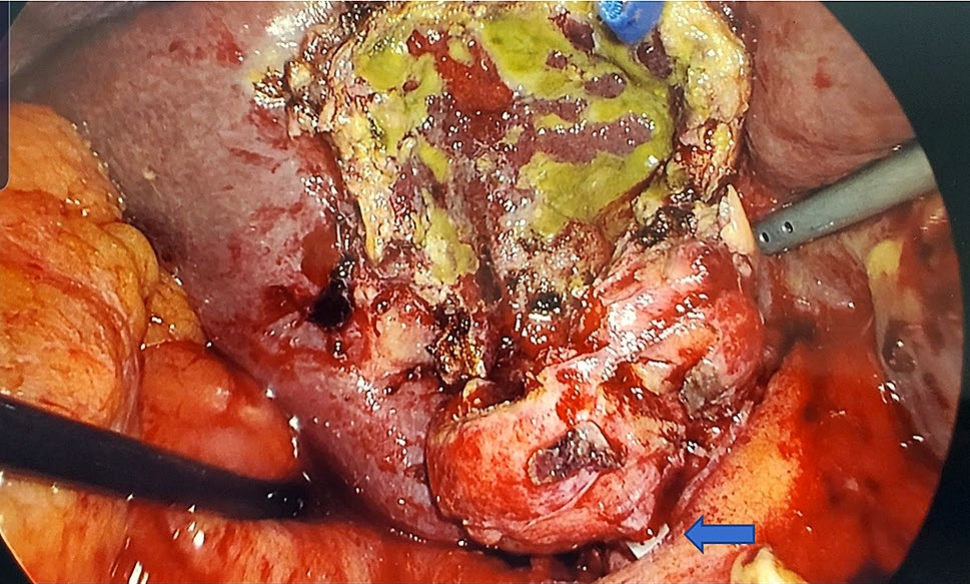
Gallbladder is incised at level of Hartmann’s pouch using a transverse incision. Hartmann’s pouch has been dissected free in a retrograde fashion towards the cystic duct. The cystic duct has been identified using the luminal view and has then been clipped guided by the luminal view (Blue Arrow). The remaining portions of Hartmann’s pouch can then be excised

In Fig. 1, retrograde dissection is performed using the luminal view until the cystic duct is reached at which point it can be controlled using an endoloop. Alternatively, as demonstrated in Fig. 2, the luminal view is used to guide cystic duct control with a hem-o-lok. The remaining portions of Hartmann’s pouch can be excised before or after cystic duct control.

## Results

Over the study period, 27 of the 587 patients (4.6%) underwent a LLC technique (median age, 60 years; M:F, 1:1.7; median ASA, 2). Pre-operative characteristics are displayed in Table 1.

**Table 1:** Pre-operative characteristics

	LLC (*n* = 27)	Non-LLC (*n* = 31)	*p*-value
Median Age (range)	60 (20–85)	65 (24–79)	0.66
M:F	1:1.7	1:2.1	0.70
Median ASA	2	2	0.75
Mode of cholecystectomy (%)			0.23
Emergency	5 (18.5)	10 (32.3)	
Elective	22 (81.5)	21 (67.7)	
Indication (%)			0.46
Cholecystitis	22 (81.5)	24 (77.4)	
Biliary Colic	2 (7.4)	2 (6.5)	
Choledocholithiasis	2 (11.1)	2 (6.5)	
Gallstone pancreatitis	0 (0.0)	3 (9.7)	
Number of previous admissions (%)			0.73
1	16 (59.3)	22 (71.0)	
2	4 (14.8)	6 (19.4)	
≥ 3	2 (7.4)	1 (3.2)	
Pre-operative intervention (%)			
ERCP	7 (25.9)	7 (22.6)	0.78
Cholecystostomy	1 (3.7)	2 (6.5)	0.64

### Peri-operative outcomes

Operative findings are displayed in Table 2. In all 27 cases, the reason for performing a LLC technique was a frozen cholecystohepatic triangle. The ‘Nassar’ difficulty grade of the LLC cases was ‘Nassar IV’ in 88.9% (24/27) and ‘Nassar V’ in the remaining 11.1% (3/27) of cases. In all cases (27/27) Hartmann’s pouch was completely excised. Cystic duct control was achieved in 85.2% (23/27). The cystic duct could not be closed in 4 cases because of either a densely adherent duodenum (2 cases) or unclear anatomy despite the LLC approach (2 cases). In three cases, inspection of the cystic duct allowed for the identification and removal of calculi before cystic duct closure.

**Table 2:** Operative findings

	LLC (*n* = 27)	Non-LLC (*n* = 31)	*p*-value
Gallbladder adhesions (%)			
Omental	12 (44.4)	11 (35.5)	0.49
Duodenal	5 (18.5)	7 (22.6)	0.70
Colonic	3 (11.1)	3 (9.7)	0.86
Gallbladder (%)			
Fibrotic gallbladder	15 (55.6)	13 (41.9)	0.30
Hartmann’s pouch stone	13 (48.1)	5 (16.1)	0.009
Empyema	7 (25.9)	8 (25.8)	0.99
Acute cholecystitis	6 (22.2)	7 (22.6)	0.97
Gangrenous gallbladder	4 (14.8)	4 (12.9)	0.83
Mucocele	4 (14.8)	4 (12.9)	0.83
Intrahepatic gallbladder	2 (7.4)	5 (16.1)	0.31
Cholecystoduodenal fistula	0 (0.0)	3 (9.7)	0.24
Cystic pedicle (%)			
Cystic pedicle impossible to dissect safely before salvage technique	27 (100)	31 (100)	1
Abnormal cystic anatomy	6 (22.2)	3 (9.7)	0.28
Hartmann’s adherent to CBD	5 (18.5)	7 (22.6)	0.70
Mirizzi’s syndrome	3 (11.1)	1 (3.2)	0.33

No bile duct injuries were sustained in the cohort (Table 3). There were three cases of haemorrhage that did not require blood transfusion. No cases of bile leak (excluding iatrogenic perforation) were detected during the procedure.

**Table 3:** Operative details and outcomes

	LLC	Non-LLC	*P* value
Median operation length [minutes (range)]	116 (67–208)	115 (46–271)	0.82
Median blood loss [mls (range)]	Minimal (minimal-600mls)	Minimal (minimal – 300 mls)	0.77
Cystic duct control achieved (%)	23 (85.2)	4 (12.9)	< 0.01
Endoloops	15 (65.2)	2 (50.0)	
Clips	6 (26.1)	2 (50.0)	
Vicryl ties	2 (8.7)	0 (0.0)	
Part of posterior wall left in situ (%)	14 (51.9)	16 (51.6)	0.82
Intra-operative complication (%)	3 (11.1)	6 (19.4)	0.39
Haemorrhage	3 (11.1)	5 (16.1)	
Bile duct injury	0 (0.0)	1 (3.2)	
Intra-operative drain inserted (%)	19 (70.4)	23 (74.2)	0.75
Stones spilled (%)	2 (28.6)	2 (6.5)	0.89

The post-operative course was unremarkable in 20 patients (74.1%) without the requirement for post-operative imaging/intervention, surgical HDU admission, post-operative complication, or readmission (Table 4). One patient was admitted to surgical HDU post-operatively for refractory hyperkalaemia. There were two post-operative collections, one of which was managed with an USS-guided drain. There were no post-operative bile leaks and no patients required a post-operative ERCP. The median post-operative length of stay was 1-day. There were 4 cases of readmission all because of abdominal pain, two of which underwent CT abdomen/pelvis scans which were unremarkable.

**Table 4:** Post-operative outcomes

	LLC (*n* = 27)	Non-LLC (*n* = 31)	*p*-value
Median post-operative length of stay, days (range)	1 (0–19)	3 (0–19)	< 0.01
Post-operative surgical HDU admission (%)	1 (3.7)	1 (3.2)	0.92
Further imaging (%)	6 (22.2)	11 (35.5)	0.27
CT abdomen/pelvis	4 (14.8)	10 (32.3)	0.12
MRCP	2 (7.4)	3 (9.7)	0.76
Further intervention (e.g. ERCP, return to theatre) (%)	1 (3.7)	8 (25.8)	0.02
ERCP	0 (0.0)	8 (25.8)	< 0.01
*I*/*R* drainage	1 (3.7)	0 (0.0)	1
Post-operative complication (Clavien–Dindo ≥ 2) (%)	2 (7.4)	12 (38.7)	< 0.01
Collection	2 (7.4)	2 (6.5)	0.89
Bile leak	0 (0.0)	11 (35.5)	< 0.01
Retained stones	0 (0.0)	2 (6.5)	0.49
AKI	0 (0.0)	3 (9.7)	0.24
Pancreatitis	0 (0.0)	1 (3.2)	1
Readmission (%)	4 (14.8)	8 (25.8)	0.11
Mortality (%)	0 (0.0)	1 (3.2)	1

### Comparison of peri-operative outcomes between LLC and non-LLC

There was no statistical difference in any of the pre-operative characteristics between the LLC and non-LLC group (*p *> 0.05) (Table 1). The LLC group had a higher rate of Hartmann’s pouch stones (*p *= 0.009) but otherwise there was no significant difference between the two groups (Table 2).

The non-LLC group (*n *= 31) was made up of 21 subtotal and 10 total cholecystectomies. The Nassar grading was IV in 27 patients (87.1%) and V in the remaining 4 patients (12.9%). Cystic duct control was achieved in 4 patients (12.9%).

In the comparison to the non-LLC group, LLC had favourable outcomes with lower rates of post-operative complication (*p *< 0.01), bile leak (*p *< 0.01), post-operative ERCP (*p *< 0.01) and shorter length of post-operative length of stay (1 day versus 3 days; *p *< 0.05) (Table 4). There were no significant differences in rates of intra-operative complications or length of procedure between LLC and non-LLC (Table 3).

## Discussion

The present paper describes a salvage technique termed the “laparoscopic lumen-guided cholecystectomy”. Twenty-seven cases are reported in patients where the critical view of safety could not be established due to a frozen cholecystohepatic triangle. In the majority of cases cystic duct control was achieved and in all cases Hartmann’s pouch was completely excised. Rates of post-operative imaging, intervention, complication and readmission are low and post-operative length of stay was short (median, 1-day). Utilising the LLC technique in cases where the cystic pedicle could not be dissected resulted in more favourable peri-operative outcomes compared to where the LLC technique had not been attempted. For example LLC patients had significantly lower rates of bile leak, ERCP and a shorter post-operative length of stay.

In our experience, gaining access to the gallbladder through Hartmann’s pouch via the LLC method allows the surgeon both to visualise the extent of the gallbladder and to delineate the anatomy of the cystic duct. With an appreciation of the cystic anatomy, cystic duct control can be achieved in the majority of cases (85.9%). Furthermore, gaining access to Hartmann’s pouch allows for Hartmann’s pouch stones to be removed which can aid dissection of the cholecystohepatic triangle. It must be mentioned that the LLC technique was employed only in difficult cholecystectomies (Nassar ≥ 4) where the procedure could not progress with the conventional CVS approach. Despite operative difficulty, gaining access to the lumen facilitated safe progression of the operation and ensured complete resection of Hartmann’s pouch with cystic duct control in the majority.

A common salvage technique is the subtotal cholecystectomy, where a gallbladder remnant is left in situ. Institutions have adopted subtotal with great success, observing a decline in the rates of iatrogenic ductal injury as the concept of subtotal has gained popularity.[5, 11, 12]. Nevertheless, these patients suffer higher rates of less severe peri-operative complications. Retained stones and bile leaks are a natural consequence of the technique, and cases of biliary fistulas and recurrent cholecystitis have been reported. As a result these patients frequently require further imaging or intervention, particularly ERCP [7, 13–15]. On the other hand, the LLC method aims to utilise the lumen to guide further dissection to achieve cystic duct control. It is hoped that this technique will reduce the risk of these post-operative complications, particularly that of bile leak and the need for ERCP. We propose that the rate of retained stones may also be reduced. In three cases, stones were removed from the cystic duct, which arguably would not be possible without removing the gallbladder remnant and gaining an adequate view of the lumen of cystic duct. Certainly, the present data suggests LLC results in lower rates of bile leak, ERCP and a shorter length of stay compared to when LLC is not attempted.

Harilingam et al. proposed a similar technique to the LLC. This was termed the “laparoscopic modified subtotal method” whereby the gallbladder is incised at the fundus and the lumen is used to facilitate safe dissection of the anterior wall towards Hartmann’s pouch [16]. Hartmann’s is then closed leaving a small gallbladder remnant. Whilst this technique is similar to the LLC method, LLC aims to achieve further retrograde dissection towards the cystic duct and aims to achieve duct control at the level of the cystic duct, not at the level of Hartmann’s pouch. This avoids the risk of both recurrent cholecystitis and the potential problems of new stones forming in the gallbladder remnant. This also avoids a completion cholecystectomy at a later stage which may be indicated but would likely be even more challenging due to further episodes of inflammation and additional adhesions [17, 18].

Fundus-first dissection offers an alternative retrograde dissection technique. Although this has been performed safely by many institutions both as a routine and salvage technique, there are concerns of the high rate of ductal injury [19–27]. The adoption of the technique has also been slow because of difficult liver retraction required to facilitate safe dissection [28]. Although, LLC dissection also uses retrograde dissection, the dissection begins from Hartmann’s pouch, not the gallbladder fundus. In the LLC technique, both liver and gallbladder retraction is maintained by the grasper positioned on the gallbladder fundus, thus liver retraction is not considered a significant concern during dissection and optimal views are achieved.

Recent literature has observed reducing rates of conversion-to-open with the adoption of the above laparoscopic salvage techniques, particularly subtotal cholecystectomy [5, 7, 11, 12]. In the present cohort, the rate of conversion-to-open was low (1.4%). This too suggests that the utilisation of minimally invasive salvage techniques are becoming more popular in face of the challenging cholecystectomy. It is hoped that the LLC offers an additional laparoscopic salvage technique that reduces the need for conversion in face of a frozen cholecystohepatic triangle.

Limitations of the study include the low sample size. Although no cases of ductal injury were noted, rates of bile duct injury in contemporary cohorts are extremely low and a very large cohort would be required to offer reassurance in this regard [1, 2]. A further limitation is low generalisability. The LLC method is a complex technique and unlikely to be adopted by general surgeons who perform cholecystectomy infrequently. All operating surgeons performing the LLC technique in this cohort were Upper GI or HPB surgeons with specialised training in cholecystectomy.

To the best of our knowledge this is the first time the lumen-guided approach has been described in the literature. Nevertheless, we anticipate that this technique has already been used widely as a salvage technique. The present data suggests that this technique can be utilised safely with good peri-operative outcomes that are superior to alternative techniques. It is hoped that the reporting of this technique will increase awareness of the technique and its advantages in face of the difficult cholecystectomy.
